# Outcome of subthalamic nucleus deep brain stimulation on long-term motor function of patients with advanced Parkinson disease

**Published:** 2017-07-06

**Authors:** Gholam Ali Shahidi, Mohammad Rohani, Mansour Parvaresh, Bahram Haghi-Ashtiani, Maryam Saeedi, Romina Rashedi, Zeynab Noori-Motlagh

**Affiliations:** 1 Department of Neurology, Rasool-e-Akram Hospital, Iran University of Medical Sciences, Tehran, Iran; 2 Department of Neurology, Firoozgar Hospital, Iran University of Medical Sciences, Tehran, Iran; 3 Student Research Committee, School of Medicine, Iran University of Medical Sciences, Tehran, Iran

**Keywords:** Parkinson Disease, Deep Brain Stimulation, UPDRS

## Abstract

**Background:** The objective of our study was to assess Unified Parkinson Disease Rating Scale (UPDRS) score in Parkinson disease (PD) patients who underwent subthalamic nucleus (STN) deep brain stimulation (DBS) 6 years after their surgery and to compare their UPDRS score 6 years after DBS with their score before surgery and 6 months after their operation.

**Methods:** In this cross sectional study which was carried out at Neurology Department of Rasool-e Akram Hospital, Tehran, Iran, affiliated to Iran University of Medical Sciences between 2008 and 2014, 37 patients with advanced PD were enrolled using non-randomized sampling method. All of the patients underwent STN DBS surgery and one patient died before being discharged, therefore; we started our study with 36 patients. The UPDRS III total score at preoperative state, 6-month follow-up and 6-year follow-up state were compared using repeated-measure analysis of variance.

**Results:** Thirty-seven patients (26 men and 10 women) with mean age of 50 ± 3 ranging from 32 to 72 years underwent STN DBS surgery. All patients were suffering from advanced PD with mean period of 11.3 ± 1.9 years. All patients except one were followed up for six months. And 14 patients (8 men and 6 women) were included in a six-year follow-up. The UPDRS score measurements before surgery, at 6-month follow-up and 6-year follow-up were 18.22 ± 2.88, 12.80 ± 3.14, 25.0 ± 11.8, respectively. Significant increase in UPDRS score was observed between the preoperative and six-year follow-up period (P < 0.001).

**Conclusion:** In conclusion, this study suggests that total UPDRS score will increase at 5 years following STN DBS and also showed that resting tremor, one of UPDRS sub-scores, will improve over time and the benefit of DBS will be persistent even after 6 years.

## Introduction

Parkinson disease (PD) is a disabling neurodegenerative disease that is characterized by resting tremor, rigidity and stooped posture and can variably affect the daily function of the patients.^1^^,^^2^ Levodopa (L-dopa) is known as the best medication for treating PD, yet this medication has many side effects such as dyskinesia if taken for a long period.^3^^,^^4^ Deep brain stimulation (DBS) is a trending surgical method that has been mostly used in the last two decades due to being less invasive and the fact that it can be performed bilaterally.^5^^-^^10^ Subthalamic nucleus (STN) stimulation is considered as a major target in PD patients.^11^ STN DBS in PD patients has shown to improve motor function and daily activities of the patients in short-term period but there is little evidence that clarifies the role of STN DBS for improving non-motor symptoms.^12^^,^^13^ Despite lack of evidence, some studies also showed that this method of surgery can have a positive effect on some of non-motor symptoms.^14^ There are studies that have shown the effectiveness of STN DBS on motor symptoms of PD patients in short-term period using Unified Parkinson Disease Rating Scale (UPDRS).^15^^-^^18^ There is limited evidence on the effectiveness of STN DBS in long-term period; however, the study on long-term effectiveness of STN DBS was done in China but it had a small sample size and it has shown improvement only in some aspects of motor function.^19^ Since there has been a lack of clinical data on long-term effectiveness of STN DBS on motor symptoms of PD patients, this study aimed to assess UPDRS score in PD patients who underwent STN DBS 6 years after their surgery. In addition we also observed the patients to detect any possible significant improvement by comparing their UPDRS score 6 years after DBS with their score before surgery and 6 months after their operation.

## Materials and Methods

In this cross sectional study which was carried out at Neurology Department of Rasool-e Akram Hospital, Tehran, Iran, affiliated to Iran University of Medical Sciences between 2008 and 2014, 37 patients with advanced PD were enrolled using non-randomized sampling method. 

All of the patients underwent STN DBS surgery and one of the patients died before being discharged from the hospital due to myocardial infarction; therefore, we started our study with 36 patients. 

Patients were included if they improved more than 30% in L-dopa challenge test and were excluded in case of having severe cardiovascular disease, uncontrolled high blood pressure, active ischemic cardiac disease, cerebrovascular accident, cancer and also if they were consuming any anticoagulant drugs.^20^ In addition to these criteria, a stable dose of L-dopa was also maintained for at least 2 months prior to the surgery. 

The study had the approval from the Institutional Review Board of Iran University of Medical Sciences (approval ID: IR.IUMS.REC1395.8821215181). Written informed consents were taken from all patients. A short-term (6 months) and a long-term (6 years) follow-up were done to evaluate and compare UPDRS score in the patients before and after surgery. 

Stereotactic surgery was performed in all of our patients. This operation was done in different steps in order to detect the best point of stimulation in STN. First step was done using stereotactic magnetic resonance imaging (MRI). The surgeons used the MR compatible frame on patient’s head and chose the best anatomical point of STN. 

The second step was done in the operating room to assess the electrophysiological activity of different nuclei by the means of tetra polar electrodes and to find the best point for inserting the permanent electrodes in STN in the operating room. This procedure was performed under local anesthesia and a trained neurologist was present to assess clinical response to DBS while operating. After determining the optimal track, the corresponding microelectrode was removed and a permanent lead was replaced. Finally, the subcutaneous pulse generator was placed in the pectoralis major muscle after few days under general anesthesia. During the weeks following the surgery, an experienced neurologist programmed the pulse generators.

Short-term follow-up was done in all of our 36 patients. However, in long-term follow-up, 22 patients were excluded from the study due to death or because they were followed up in another center and long-term follow-up was only done in 14 patients.

Postoperative motor performance was evaluated using the UPDRS part III right after the surgery, 6 months after the surgery and also 6 years after the operation.

UPDRS part III measures items such as speech, facial expression, resting tremor, acting tremor, rigidity, finger tap, hand movement, rapid alternating movements, leg agility, rising from the chair, gait, bradykinesia, and posture stability. Each score is from 0 to 4.^21^ Lower scores show better performance.

Preoperative UPDRS III evaluation was also done in the on- and off-medication state. Postoperative scores were only assessed in on-medication state 6 months and 6 years after the surgery.

In this study, all data were analyzed by SPSS software (version 22, IBM Corporation, Armonk, NY, USA). Descriptive analysis of the data with normal distribution is available as means and standard deviation. 

The UPDRS III total score at preoperative state, 6-month follow-up and 6-year follow-up state were compared using repeated-measure analysis of variance (ANOVA). The distribution of UPDRS III sub-scores was not normal; therefore, we compared them using the Wilcoxon signed-rank test. We also used repeated-measure ANOVA to compare doses of L-dopa in three phase of our follow-up.

## Results

Thirty seven patients (26 men and 10 women) with the mean age of 50 ± 3 ranging from 32 to 72 years underwent STN DBS surgery. All patients were diagnosed with advanced PD with the mean period of 11.3 ± 1.9 years from the onset of the symptoms till the time of surgery. All patients except one, who died from the myocardial infarction, were followed up for six months, and 14 patients (8 men and 6 women) were included in a six-year follow-up. 

The UPDRS score measurements were 18.22 ± 2.88, 12.80 ± 3.14, 25.0 ± 11.8 before the surgery, 6 months, and six years after the surgery, respectively which showed significant difference between preoperative score and the score 6 years after the operation (P = 0.033), and also significant increase in UPDRS score was observed between the preoperative and six-year follow-up period (P < 0.001) (Table 1).

Sub-score measurements revealed significant differences between preoperative and six-year follow-up scores of resting tremor (P = 0.020), speech (P = 0.007), rapid alternating movements of the hands (P = 0.011), hand movements (P = 0.010), finger tap (P = 0.009), and facial expression (P = 0.021) (Table 1). Furthermore, significant differences were seen between sub-scores of speech (P = 0.007), rigidity (P = 0.022), rapid alternating movements of the hands (P = 0.003), leg agility (P = 0.028), hand movements (P = 0.005), finger tap (P = 0.001) and facial expression (P = 0.021) between the time points of 6-month and six-year follow-up (Table 1).

We also measured the doses of L-dopa, which our patients were consuming before surgery, in short-term follow-up and in long-term period.

**Table 1:** Unified Parkinson Disease Rating Scale (UPDRS) scores and sub-scores at different time points and reporting P

**Time**	**Preoperation**	**Postoperation**	**6 years after ** **surgery**	**Before ** **surgery ** **(P)**	**6 months ** **after ** **surgery ** **(P)**
**Scores**					
UPDRS (mean ± SD)	18.22 ± 2.88	12.80 ± 3.14	25.00 ± 11.80	0.033	< 0.001
Sub-scores (mean ± SD)					
Speech	1.10 ± 0.70	1.05 ± 0.89	1.90 ± 1.07	0.021	0.021
Facial expression	1.10 ± 0.40	0.97 ± 0.44	1.50 ± 0.75	0.020	0.112
Tremor at rest	1.50 ± 1.90	0.83 ±1.38	0.14 ± 0.50	0.257	0.414
Action or postural tremor of hands	0.54 ± 0.77	0.25 ± 0.50	0.28 ± 0.61	0.505	0.022
Rigidity	3.40 ± 2.60	1.40 ± 2.00	4.20 ± 4.20	0.009	0.001
Finger taps	1.60 ± 1.30	1.25 ± 1.60	3.40 ± 1.90	0.010	0.005
Hand movements	1.41 ± 1.40	1.02 ± 1.44	2.60 ± 1.70	0.011	0.003
Rapid alternating movements of hands	0.83 ± 1.05	0.50 ± 1.02	2.60 ± 1.70	0.077	0.028
Leg agility	2.80 ± 1.70	2.25 ± 1.90	3.50 ± 2.10	0.157	0.206
Arising from chair	0.19 ± 0.40	0.16 ± 0.50	0.50 ± 0.65	0.317	0.132
Posture	0.83 ± 0.50	0.70 ± 0.60	0.92 ± 0.80	0.194	0.053
Gait	0.75 ± 0.69	0.60 ± 0.70	1.00 ± 0.80	0.160	0.190
Postural stability	1.02 ± 0.65	1.00 ± 0.70	1.40 ± 0.85	0.132	0.366
Body bradykinesia and hypokinesia	0.91 ± 0.70	0.63 ± 0.79	1.00 ± 0.87	0.538	0.111

SD: Standard deviation; UPDRS: Unified Parkinson Disease Rating Scale

**Table 2:** L-dopa dosage in patients at different time points

**Follow-up periods**	**Number of patients**	**Gender**	**Age (year) ** **(mean ± SD)**	**L-dopa (mg/dl) ** **(mean ± SD)**
Preoperation (baseline)	36	Men: 26, Women: 10	50 ± 3	1269 ± 114
Six-month (short-term)	36	Men: 26, Women: 10	50 ± 3	783 ± 87
Six-year (long-term)	14	Men: 8, Women: 6	49 ± 8	992 ± 170

The mean L-dopa equivalent doses showed significant decline from 1296 ± 224 mg/d before surgery (baseline) to 783 ± 87 mg/d after DBS in short-term period (P < 0.005), but there was no significant difference between the doses of L-dopa consumption in long-term follow-up and short-term follow-up (P = 0.110). Furthermore, there was no significant decline between long-term follow up and preoperative doses of L-dopa (P = 0.530) (Table 2).

## Discussion

The purpose of this study was to assess UPDRS score in patients suffering from PD who underwent STN DBS 6 years ago and to compare UPDRS score 6 years after the surgery with their score 6 months after the surgery and also their score before the STN DBS. We measured their UPDRS score while they were all on medication and according to their UPDRS score after 6-year follow-up, we found that total motor function got worse after 6 years following the operation compared to the score before and 6 months after the surgery. We also compared UPDRS sub-scores in patients 6 years after the surgery with their score in 6-month follow-up and also their pre-operative sub-scores. We found a significant improvement in patients’ resting tremor in 6-year follow-up compared to their pre-operative state. We also observed an improvement in the tremor at rest of patients 6 years following the surgery in comparison to their score in 6-month follow-up, although it was statistically insignificant. Total worsening in motor function of the patients in 6-year follow-up compared to their state before surgery was mainly due to their poor score in speech, facial expression, finger taps, hand movements and rapid alternating movements of hands. When we compared sub-scores 6 years after STN DBS with patients’ sub-scores 6 months after the surgery, we found that the reason for worse motor status was mainly the poor result in speech, facial expression, rigidity, finger taps, hand movements and rapid alternating movements of hands and leg agility. 

We also measured the dosage of L-dopa needed in patients 6 months and 6 years after the surgery and we compared them with the daily doses of the medication needed before the operation and we found that during short-term follow-up, patients’ requirement for L-dopa had significantly decreased. However, in a long-term, dose of L-dopa needed in patients had reduced, but it was statistically insignificant.

In a study that was conducted in 2009 on the same patients as the current study, UPDRS score was measured 6 months after STN DBS and the results indicated a significant improvement in UPDRS score in short-term follow-up.^22^ In some studies which observed motor symptoms of the patients in 5 years and a few studies which followed the patients more than 8 years, DBS was shown to be beneficial for tremor, rigidity, and bradykinesia but not for axial symptoms.^8^^,^^23^^-^^27^ However, in our study, the efficacy of STN DBS was only persistent for tremor at rest and not for other symptoms.

In another study that was conducted in China, it was shown that in the off-medication state, motor symptoms were significantly improved 5 years following the operation. However, similar to our study, it was implied that with longer follow-up in on-medication state, worsening of motor function is expected due to L-dopa resistance symptoms.^19^

One of our study limitations was small sample size, which could inevitably result in declined statistical power. Furthermore, we lost 61.1% of our patients (22 from 36 patients) in 6-year follow-up and that could also lead to bias. Finally, we could not evaluate UPDRS in off-medication state since patients were unwilling to discontinue their L-dopa.

## Conclusion

In conclusion, this study suggests that total UPDRS score will increase at 5 years following STN DBS and the patients’ motor function will worsen in log-term follow-up after the operation. Our study also showed that resting tremor, one of UPDRS sub-scores, will improve over time and the benefit of DBS will be persistent even after 6 years.

## Notes:

**How to cite this article:** Shahidi GA, Rohani M, Parvaresh M, Haghi-Ashtiani B, Saeedi M, Rashedi R, et al. Outcome of subthalamic nucleus deep brain stimulation on long-term motor function of patients with advanced Parkinson disease. Iran J Neurol 2017; 16(3): 107-11.
